# Machine Learning Classifiers for Endometriosis Using Transcriptomics and Methylomics Data

**DOI:** 10.3389/fgene.2019.00766

**Published:** 2019-09-04

**Authors:** Sadia Akter, Dong Xu, Susan C. Nagel, John J. Bromfield, Katherine Pelch, Gilbert B. Wilshire, Trupti Joshi

**Affiliations:** ^1^Informatics Institute, University of Missouri, Columbia, MO, United States; ^2^Electrical Engineering and Computer Science, University of Missouri, Columbia, MO, United States; ^3^Christopher S. Bond Life Sciences Center, University of Missouri, Columbia, MO, United States; ^4^OB/GYN and Women’s Health, University of Missouri School of Medicine, Columbia, MO, United States; ^5^Boone Hospital Center, Columbia, MO, United States; ^6^Health Management and Informatics, University of Missouri, Columbia, MO, United States

**Keywords:** endometriosis, machine learning, classification, methylomics, transcriptomics, DNA methylation, RNA-seq, translational bioinformatics

## Abstract

Endometriosis is a complex and common gynecological disorder yet a poorly understood disease affecting about 176 million women worldwide and causing significant impact on their quality of life and economic burden. Neither a definitive clinical symptom nor a minimally invasive diagnostic method is available, thus leading to an average of 4 to 11 years of diagnostic latency. Discovery of relevant biological patterns from microarray expression or next generation sequencing (NGS) data has been advanced over the last several decades by applying various machine learning tools. We performed machine learning analysis using 38 RNA-seq and 80 enrichment-based DNA methylation (MBD-seq) datasets. We experimented how well various supervised machine learning methods such as decision tree, partial least squares discriminant analysis (PLSDA), support vector machine, and random forest perform in classifying endometriosis from the control samples trained on both transcriptomics and methylomics data. The assessment was done from two different perspectives for improving classification performances: a) implication of three different normalization techniques and b) implication of differential analysis using the generalized linear model (GLM). Several candidate biomarker genes were identified by multiple machine learning experiments including *NOTCH3*, *SNAPC2*, *B4GALNT1*, *SMAP2*, *DDB2, GTF3C5*, and *PTOV1* from the transcriptomics data analysis and *TRPM6*, *RASSF2*, *TNIP2*, *RP3-522J7.6*, *FGD3*, and *MFSD14B* from the methylomics data analysis. We concluded that an appropriate machine learning diagnostic pipeline for endometriosis should use TMM normalization for transcriptomics data, and quantile or voom normalization for methylomics data, GLM for feature space reduction and classification performance maximization.

## Introduction

Endometriosis is a complex and common gynecological disorder, and the etiology is poorly understood (Halme et al., 1984). The impact of endometriosis is very high. About 176 million women worldwide and about 8.5 million women in North America suffer from endometriosis (David Adamson et al., 2010). Five to ten percent of women who are of reproductive age, 20–30% of women with subfertility, and 40–60% of women with chronic pelvic pain and infertility are suffering from endometriosis (Selçuk and Bozdağ, 2013). Nearly 70% of teens with pelvic pain are later diagnosed with endometriosis (Yeung et al., 2011). Endometriosis is a leading cause of the 600,000 hysterectomies performed in the US every year (Burkett et al., 2011) and significantly impairs mental and physical quality of life in patients. Moreover, work performance for women with endometriosis is seriously compromised. Endometriosis causes a large economic burden due to loss of workdays and the health-care costs due to outpatient visits, hospitalization, and medications, which in the US have been estimated to be $22 billion each year (Simoens et al., 2007).

A gold standard for endometriosis diagnostic approach is laparoscopy, which is an invasive procedure. Due to the lack of definitive clinical diagnostic symptoms and an easy-to-perform molecular diagnostic approach, current diagnostic latency is on average 4 to 11 years (Agarwal et al., 2019). Therefore, early intervention is crucial for reducing suffering and expenses related to the disease. A minimally invasive diagnostic approach, such as endometrial biopsy, would be very useful for reducing diagnostic latency. Endometriosis patients have an altered methylome (DNA methylation) and transcriptome (RNA-seq), and these differences in DNA methylation and gene expression could lead to the identification of biomarkers for developing a minimally invasive diagnostic technique for endometriosis (Eyster et al., 2007; Wu et al., 2007; Xue et al., 2007a; Xue et al., 2007b; Lee et al., 2009). In a DNA microarray study comparing eutopic endometrium and ectopic endometrium suggested that alterations of cell adhesion-associated genes may contribute to the adhesive and invasive properties of ectopic endometrium (Eyster et al., 2007). In a mouse model of endometriosis with bisulphite-based DNA methylation suggested that significant changes occur in multiple markers of endometrial receptivity in the eutopic endometrium after induction of endometriosis (Lee et al., 2009). A cross-sectional measurement of gene expression levels of *DNMT1*, *DNMT3A*, and *DNMT3B* on endometriotic tissue demonstrated that those genes were overexpressed in the ectopic endometrium as compared with normal control subjects or the eutopic endometrium of women with endometriosis (Wu et al., 2007). Also, differential methylation of a CpG island at the ESR2 promoter region (Xue et al., 2007a) and SF-1 promoter and exon I regions (Xue et al., 2007b) may be key mechanisms related to endometriosis.

Discovery of relevant biological patterns from microarray expression data or next generation sequencing data have been advanced over the last several decades by applying various machine learning tools (Tarca et al., 2007; Liu et al., 2013; Neelima and Prasad Babu, 2017). Both unsupervised and supervised machine learning methods have been applied widely on microarray expression data (Vandesompele et al., 2002; Libbrecht and Noble, 2015). In the unsupervised machine learning application, some studies evaluated the clustering techniques such as hierarchical clustering and K-means clustering for identifying the groups of genes that share similar functions or expressions (Mudge et al., 2013; GTEx Consortium, 2015; Melé et al., 2015). For the application of supervised machine learning methods, some studies evaluated the application of disease vs. healthy classification tasks using various methods such as decision trees, random forests, artificial neural networks (ANN), support vector machines (SVM), and Bayesian networks (Pirooznia et al., 2008). Availability of both transcriptomics and methylomics data have greatly increased in recent years, which created the opportunity for using those data in clinical diagnostics (Mikeska et al., 2012; Byron et al., 2016). Unlike microarray gene expression data, application of machine learning classifiers on transcriptomics or methylomics data have been limited with various success (Bhasin et al., 2005; Wei et al., 2006; Bock, 2012; Cai et al., 2015; Thompson et al., 2016; Johnson et al., 2018). The difference of gene expressions in transcriptomics data or the difference of DNA methylation in methylomics data can provide avenues for the development of endometriosis diagnostic method (Eyster et al., 2007; Wu et al., 2007; Xue et al., 2007a; Xue et al., 2007b; Lee et al., 2009). In this work, we assess various supervised machine learning methods trained on both transcriptomics and methylomics data for classifying endometriosis samples from the control for creating highly accurate diagnostic predictive models.

An earlier work evaluated the performance of classification models using transcriptomics data (Akter et al., 2018). This work aims to systematically examine how well various state-of-the art supervised machine learning methods perform in classifying endometriosis and control samples using both transcriptomics and methylomics data. The assessment was done from three different perspectives: a) implication of three different normalization techniques on prediction performances, b) implication of differential analysis on prediction performances. In addition, network and functional enrichment analysis was conducted using the genes identified from different machine learning models.

## Materials and Methods

### Subjects and Tissue Collection

Subjects for the study were aged between 18 and 49 years and all undergoing a laparoscopy procedure—either diagnostic laparoscopy for pain or infertility or seeking laparoscopic sterilization. Prior to surgery, the physician obtained informed consent following the IRB protocol. Endometrial biopsies, which yield ≥250 mg of tissue, were collected using suction pipelles (Cooper Surgical Uterine Explora Model I) under general anesthesia prior to surgery. Endometrial biopsy is a quick, minimally invasive procedure, lasting ≤5 min, with minimal risk of infection, uterine perforation, or bleeding. During laparoscopy, the physician thoroughly examined the peritoneal cavity and visually confirmed the presence or absence of endometriosis. If present, at least one endometriotic lesion was sent to pathology for histological confirmation of endometriosis. Endometriosis patients had visually and histologically confirmed endometriosis.

**Table 1** presents the inclusion and exclusion criteria for the two populations. Samples were collected from three different institutes: 1) Women’s and Children’s Hospital, University of Missouri; 2) Boone Hospital, Columbia, MO; and 3) University of California, San Francisco. The tissue samples were processed for generating high-throughput mRNA (RNA-Seq) data and enrichment-based DNA methylation (MBD-seq) data using the Illumina Next Seq NGS technology. Our transcriptomics dataset includes 38 single-end RNA-seq samples (22 controls and 16 endometriosis). The methylomics dataset includes 80 enrichment-based DNA methylation samples (36 controls and 44 endometriosis) where 77 (35 controls and 42 endometriosis) met the quality control criteria.

**Table 1:** Inclusion and exclusion criteria.

Group	Inclusion criteria	Exclusion criteria
Controls	Age 18 to 49 years	Visual observation of lesions
Endometriosis	Age 18 to 49 years Laparoscopic and pathology confirmed	Diagnostic laparoscopy without visual observation of endometriotic lesions

### Transcriptomics and Methylomics Data Preprocessing Workflow

We preprocessed our data using several widely accepted bioinformatics tools. The transcriptomics dataset was processed in five steps, and the methylomics dataset was processed in seven steps. Steps 1 to 3 were same for both datasets. In the first step, all raw data were checked for quality control using FastQC (Andrews). In the second step, Cutadapt (Martin, 2011) was used to remove reads with low-quality bases, adapter sequences, and other contaminating sequences. In step three, Bowtie2 (Langmead et al., 2009) was applied to align sequence reads to the reference genome *hg38*. In the fourth step for RNA-seq, TopHat (Trapnell et al., 2009) was used to discover the locations of short sequence reads with respect to the reference. In step five for RNA-seq, HTSeq (Anders et al., 2015) was applied to generate the read count data, which was then filtered to remove very low count genes. The filtering criterion was to keep the genes that have at least 1 count per million (cpm) reads mapped in at least *n* samples where *n* is the smallest group size. In the fourth step for DNA methylation, each sample’s read was aligned against the reference genome hg38 using Bowtie2 (Langmead et al., 2009). In the fifth step for DNA methylation, we used Samtools (Li et al., 2009) and Picard (Picar) for sorting and removing duplicate reads. In the sixth step for DNA methylation, we segmented the genome sequence into 1,000 bases tiling windows, which is widely used. The seventh step for DNA methylation is to record the number of reads that are mapped to each methylated region. Read counts are the number of aligned reads that uniquely map to the hg38 reference genome. Several R packages (MEDIPS, BSgenome, BSgenome.Hsapiens.UCSC.hg38) were applied to generate the read count data, which was then filtered to remove very low count methylated regions. The filtering criterion was to keep the regions that have nonzero counts per million (cpm) reads mapped in at least *n* samples where *n* is the smallest group size. A study on comparing normalization techniques in RNA-seq analysis demonstrated that normalization methods have impacts on the results (Lin et al., 2016). In this study, the read count data were normalized using three different techniques: a) logarithm of counts per million (logCPM) of trimmed mean of M values (TMM) (Smyth, 2004), b) quantile normalization (qNorm) (Bolstad et al., 2003), and c) Voom normalization (vNorm) (Smyth, 2004).

In the methylomics data analysis, our goal is to identify the methylated regions of interest (MROI) and find the nearby genes. Mapping of an MROI to the reference annotation information helped us to extract the nearest genes from that MROI. Our goal is to identify the genomic features such as the protein-coding genes, long intergenic noncoding RNA (lincRNA) genes, microRNA (miRNA) genes, ribosomal ribonucleic acid (rRNA) genes, small nucleolar RNA (snoRNA) genes, and small nuclear RNA (snRNA) genes. The distance threshold for the MROI position to the genomic region was set to 10,000 bp.

### Differential Analysis

To identify the differentially expressed genes (DEGs) in the transcriptomics dataset or the differentially methylated regions (DMRs) in the methylomics dataset between the control and endometriosis cases, a generalized linear model (GLM) was applied followed by likelihood ratio test using the edgeR (Robinson et al., 2010) package. The trimmed mean of M values (TMM) normalization was performed to normalize read counts among different samples. The significance of the genes was defined by using an adjusted p-value cutoff set at 5% using the false discovery rate (FDR) method for multiple testing (Benjamini and Hochberg, 1995).

### Network and Functional Enrichment Analysis

We used the GeneMANIA (Montojo et al., 2014) application in Cytoscape (Shannon et al., 2003) for the network analysis and functional enrichment analysis. For a given gene list, GeneMANIA can build a weighted functional interaction network using a database of almost 2,300 networks. The networks are organized into different groups such as co-expression, physical interaction, genetic interaction, shared protein domains, co-localization, pathway, etc. To generate a network, Pearson correlation is used as the degree of interaction strength between each pair of genes. Utilizing the publicly available datasets, the GeneMANIA algorithm can predict genes or gene products that are highly related to the original gene list. Hypergeometric test was applied for the functional enrichment analysis with q-values cutoff of 0.10 using the Benjamini–Hochberg procedure.

### Machine Learning Classifiers

#### Decision Tree

Decision tree is a tree-based algorithm that can be described as IF–THEN rules (Quinlan, 1993). There are many varieties of the decision tree algorithm based on the attribute selection criteria such as Iterative Dichotomiser 3 (ID3) (Quinlan, 1986), Classification and Regression Trees (CART) (Breiman et al., 1984), and C4.5 (Quinlan, 1993). Decision tree is constructed in two phases. First, a large tree is grown to fit the data closely. Second, the tree is pruned by removing parts that are predicted to have a relatively high error rate. C4.5 is a popular algorithm for decision tree construction that uses entropy minimization or information gain for attribute selection criteria. We used an improved version of C4.5 called C5.0/see5 (Data Mining Tools See5 and C5.0) for constructing the decision tree in this study. Confidence factor is used as a parameter for tree pruning in C5.0. The default value for confidence factor is 25% or 0.25. If the value of confidence factor is smaller than 0.25, it causes more pruning and *vice versa*.

#### Biosigner

Biosigner is an enhanced algorithm for detecting biomarkers (Rinaudo et al., 2016). The Biosigner algorithm includes four steps: 1) bootstrap resampling (default is boot = 50), 2) feature ranking, 3) selection of significant features called signature set, and 4) building the final model that is restricted to the features from the signature set. In Biosigner, three different machine learning classifier algorithms [partial least squares discriminant analysis (PLSDA) (Wold et al., 2001; Barker and Rayens, 2003), random forest (RF) (Breiman, 2001), and support vector machine (SVM) (Boser et al., 1992)] were used for constructing three different models. As the input to the Biosigner algorithm, we used the TMM normalized data as expression data.

### Machine Learning Experimental Approach

We performed six different experiments using the decision tree classifier (see **Table 2**). Performance measures of each model were computed using the cross-validation approach described previously. We used the default value of confidence factor (0.25) so that the decision tree is optimally pruned. GLM was applied on the whole dataset for the decision tree experiments.

**Table 2:** Machine learning experimental approach using decision tree.

Experimental name	Normalization	GLM	Decision tree
TMM + Decision Tree	TMM		X
qNorm + Decision Tree	qNorm		X
vNorm + Decision Tree	vNorm		X
TMM + GLM + Decision Tree	TMM	X	X
qNorm + GLM + Decision Tree	qNorm	X	X
vNorm + GLM + Decision Tree	vNorm	X	X

For testing with the Biosigner algorithm, we used TMM as the expression data. The TMM data were then used for constructing the classifier models (PLSDA, random forest, and SVM) using Biosigner. We used the default value for the boot parameter, which is 50. In some iterations of the leave-one-out cross-validation, Biosigner was unable to produce a prediction result for the test record using the predict function that uses the signature model, if the signature model was not produced in those iterations. In such a scenario, the model with tier A was used for prediction.

The dataset were filtered for low read count genes for the transcriptomics datasets and for low read count methylated regions for the methylomics dataset. For the transcriptomics dataset, the experiments were conducted in two scenarios: a) all genes including protein-coding, lincRNA gene, miRNA gene, rRNA gene, etc. are present in the dataset after removing the genes with lower read counts, and b) only protein-coding genes are present in the dataset after removing the genes with lower read counts. For the methylomics dataset, all methylated regions except lower read counts were present.

### Cross-Validation and Model Performance

For model validation and comparing results between the methods, we applied the leave-one-out cross-validation for computing the performance measures. This ensures two things: 1) the record used for model validation is not used for model construction, and 2) all records are used for model validation. This technique is useful for dataset with smaller number of samples such as in our study. The final model is constructed using all records. We computed several model performance measures: accuracy, sensitivity, specificity, precision, F_1_ score, Matthews correlation coefficient (MCC), and area under the receiver operating characteristics curve (AUC); the leave-one-out cross-validation approach was used for calculating these measures.

## Results

### Data Preprocessing and Differential Analysis

There were a total of 38 samples in the transcriptomics dataset. After preprocessing of the RNA-seq data of each samples, we created a dataset containing the read counts of 58,050 genes in which 18,852 genes were protein-coding. After applying the filtering criteria for low count genes, 14,154 genes were included in the dataset in which 11,687 of them were protein-coding genes. We performed differential analysis using the GLM followed by likelihood ratio test on 14,154 genes and found 28 DEGs: 5 upregulated and 23 downregulated genes. We also performed the differential analysis on the 11,687 protein-coding genes only and found 11 protein-coding DEGs: one upregulated and 10 downregulated genes.

In the methylomics dataset, out of the 80 samples, 77 samples met the quality control criteria (35 controls and 42 endometriosis). After preprocessing of the enrichment-based DNA methylation (MBD-seq) data of each sample, we created a dataset containing the read counts of 3,088,281 methylated regions. After applying the filtering criteria for lower read counts, 2,577,382 methylated regions were included in the dataset. We performed the differential analysis using GLM on 2,577,382 methylated regions and found 365 DMRs in which 303 of them were hypermethylated and 62 of them were hypomethylated.

### Decision Tree Results Using Transcriptomics Data

**Figure 1:**
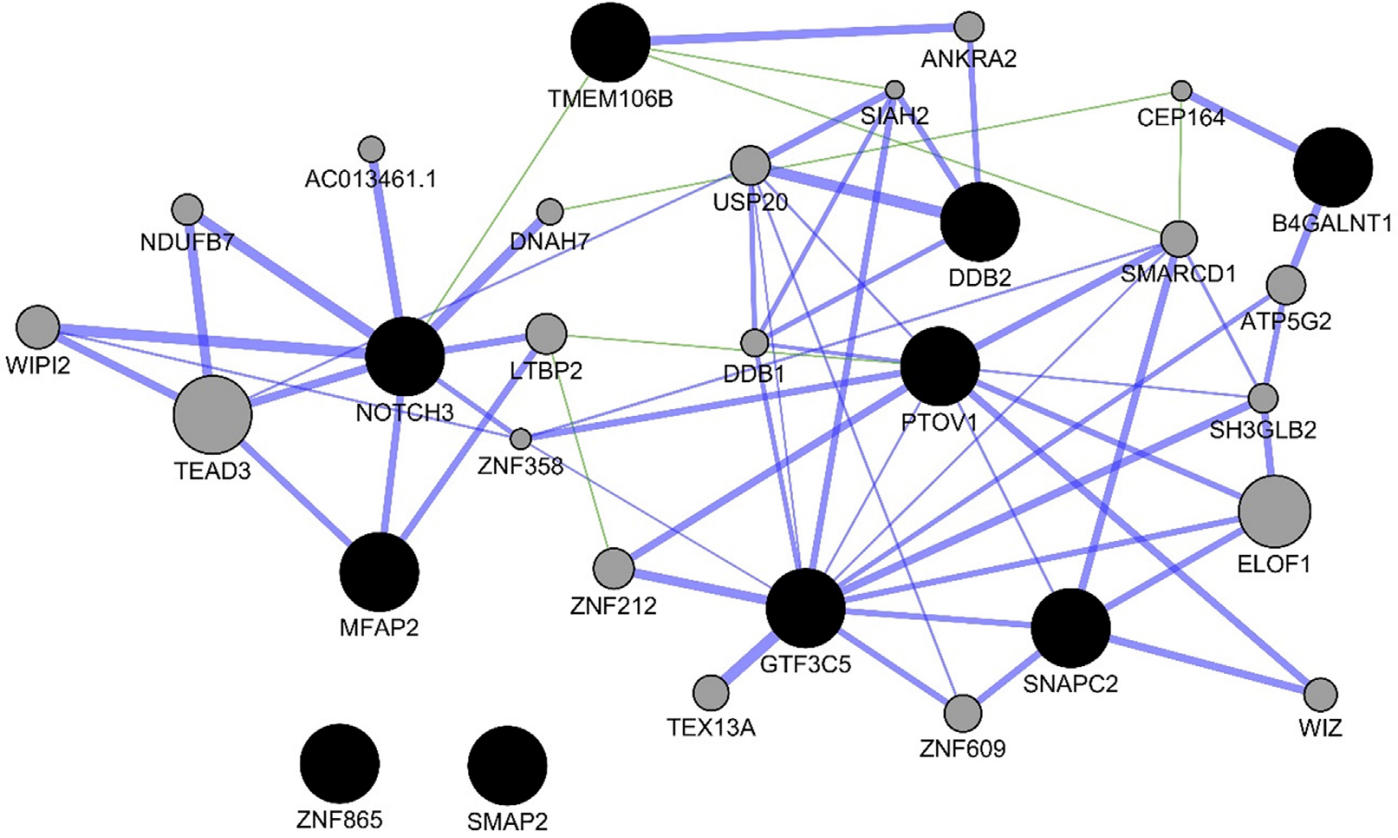
Gene interaction network among the genes from the decision tree models using the protein-coding genes of transcriptomics data. Black circles denote the candidate biomarkers genes, and gray circles denote the GeneMANIA-predicted genes. Blue and green edges represent co-expression (network weight 99.70) and genetic interactions (network weight 0.33), respectively.

For the experiments using both the protein-coding and nonprotein-coding genes (denoted as “all genes” in this article), we used 14,154 genes in these experiments. For the experiments using only the protein-coding genes, we used only the protein-coding genes that include 11,687 genes. We applied the six experiments (separately for all genes and protein-coding genes) using the decision tree algorithm. **Table 3** presents the decision tree models from those experiments. A total of 13 candidate biomarker genes were identified from the transcriptomics experiments using decision tree. Among those genes, eight genes were differentially expressed (seven downregulated and one upregulated). Six genes were identified from the models using all genes in which three of them are protein-coding. Nine genes were identified from the models using protein-coding genes. Two genes (*NOTCH3* and *TMEM106B*) were found common between the two groups of results (all vs. protein-coding genes). *NOTCH3* was present in all models, and *KLF2P1* was present in three models using decision tree. “TMM + GLM + Decision Tree” experiments (all vs. protein-coding genes) achieved the best performances (see Performance Evaluation section for more details). **Figure 1** presents the gene interaction network among all the genes from different decision tree models of the transcriptomics dataset. Most of the query genes and predicted genes are linked by co-expression network with weight 99.70. There were no significant GO annotation from the functional enrichment analysis of these genes.

**Table 3:** Decision tree models using transcriptomics data.

Gene feature set	Experiment name	Tree model
All	TMM + Decision Tree	NOTCH3 <= 0.3994181: endometriosis (10) NOTCH3 > 0.3994181: :...TMEM106B <= 2.207379: control (23/1) TMEM106B > 2.207379: endometriosis (5)
All	qNorm + Decision Tree	NOTCH3 <= 1.710526: endometriosis (13/1) NOTCH3 > 1.710526: :... RP11-792A8.1<= 1.052632: control (22/1) RP11-792A8.1> 1.052632: endometriosis (3)
All	vNorm + Decision Tree	NOTCH3 <= -0.05049461: endometriosis (13/1) NOTCH3 > -0.05049461: :...RP11-459F6.3 <= 1.434793: endometriosis (3) RP11-459F6.3 > 1.434793: control (22/1)
All	**TMM + GLM + Decision Tree**	NOTCH3 <= 0.3994181: endometriosis (10) NOTCH3 > 0.3994181: :...KLF2P1 > 3.247368: endometriosis (4) KLF2P1 <= 3.247368: :...MFAP2 <= -0.1358892: endometriosis (3/1) MFAP2 > -0.1358892: control (21)
All	qNorm + GLM + Decision Tree	NOTCH3<= 1.684211: endometriosis (13/1) NOTCH3> 1.684211: :... SMAP2<= 17.94737: control (22/1) SMAP2> 17.94737: endometriosis (3)
All	vNorm + GLM + Decision Tree	NOTCH3 <= -0.05049461: endometriosis (13/1) NOTCH3 > -0.05049461: :...KLF2P1 <= 3.057986: control (22/1) KLF2P1 > 3.057986: endometriosis (3)
Protein-Coding	TMM + Decision Tree	NOTCH3 <= 1.644335: endometriosis (10) NOTCH3 > 1.644335: :...TMEM106B <= 3.460871: control (23/1) TMEM106B > 3.460871: endometriosis (5)
Protein-Coding	qNorm + Decision Tree	NOTCH3 <= 1.894737: endometriosis (13/1) NOTCH3 > 1.894737: :...SMAP2 <= 1: control (22/1) SMAP2 > 1: endometriosis (3)
Protein-Coding	vNorm + Decision Tree	NOTCH3 <= 1.293087: endometriosis (13/1) NOTCH3 > 1.293087: :...DDB2 <= 5.616763: endometriosis (3) DDB2 > 5.616763: control (22/1)
Protein-Coding	**TMM + GLM + Decision Tree**	NOTCH3 <= 1.641844: endometriosis (10) NOTCH3 > 1.641844: :...B4GALNT1 <= 7.888268: endometriosis (3) B4GALNT1 > 7.888268: :...ZNF865 <= 1.835846: endometriosis (2) ZNF865 > 1.835846: control (23/1)
Protein-Coding	qNorm + GLM + Decision Tree	NOTCH3 <= 1.815789: endometriosis (13/1) NOTCH3 > 1.815789: :...PTOV1 > 779.6053: control (17) PTOV1 <= 779.6053: :...GTF3C5 <= 0.8157895: endometriosis (4) GTF3C5 > 0.8157895: control (4)
Protein-Coding	vNorm + GLM + Decision Tree	NOTCH3 <= 1.293087: endometriosis (13/1) NOTCH3 > 1.293087: :...SNAPC2 -0.7898067: endometriosis (2) SNAPC2 > -0.7898067: control (23/2)

The best model in each subgroup of experiment is presented in bold text.

### Biosigner Results Using Transcriptomics Data

**Figure 2:**
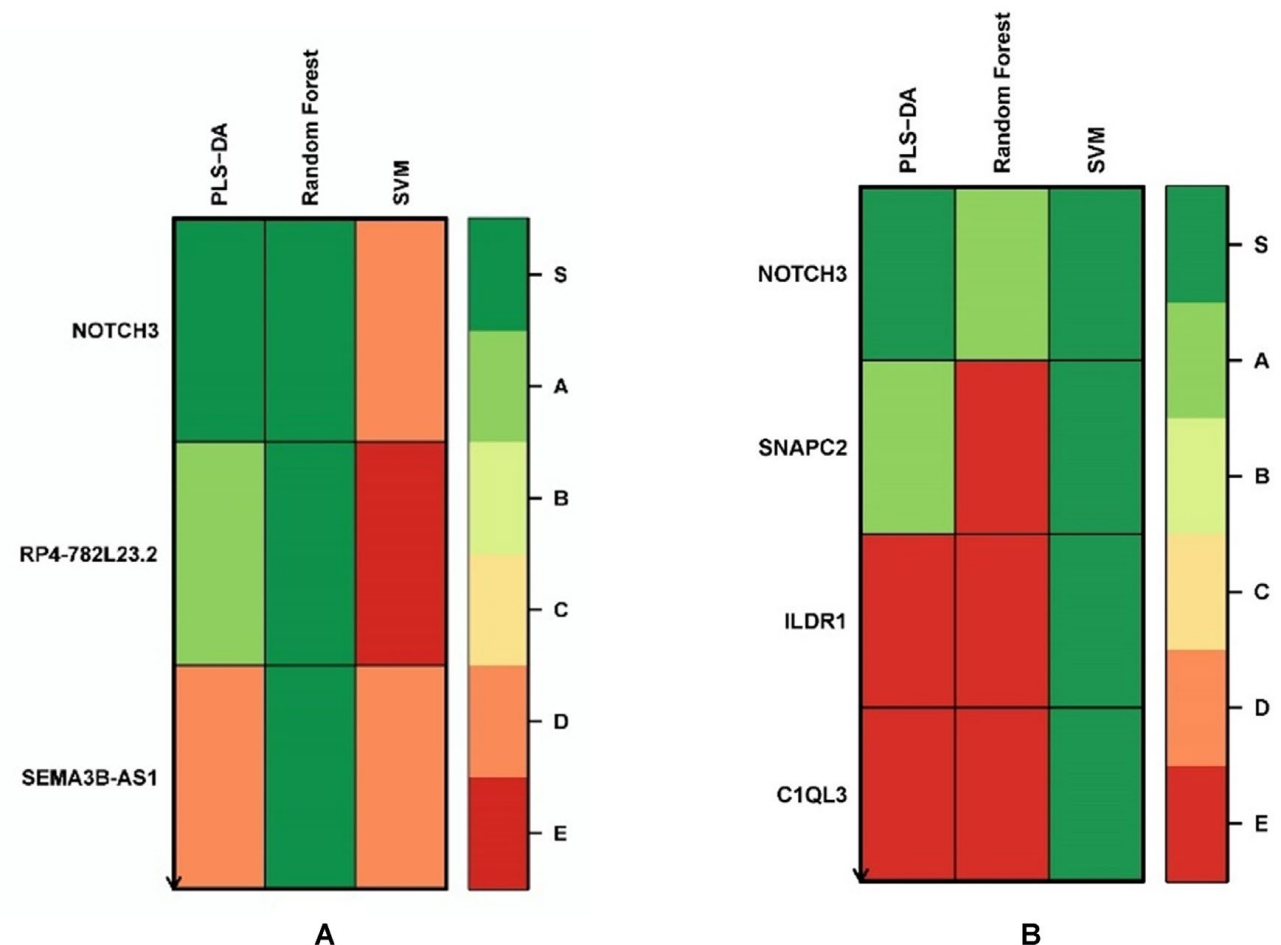
Gene tier plot from Biosigner using transcriptomics data from the experiments using: **(A)** all genes, **(B)** protein-coding genes only; S, signature genes; A–E=A is a higher tier, and E is a lower tier.

We applied the Biosigner algorithm on 14,154 genes including both the protein-coding and nonprotein-coding genes (denoted as “all genes” in this article) and on 11,687 protein-coding genes only, separately. As the input of the Biosigner algorithm, we used the TMM normalized data as the expression data. The Biosigner algorithm constructed three different models: PLSDA, random forest, and SVM. **Figure 2A** presents the gene tier plot (S = signature genes; A–E = A is a higher tier gene and E is a lower tier genes). Biosigner identified three genes as the potential set of biomarkers: *NOTCH3*, *RP4-782L23.2*, and *SEMA3B-AS1* using all genes. **Figure 2B** presents the gene tier plot of four genes as the potential set of biomarkers identified by Biosigner: *NOTCH3*, *SNAPC2*, *ILDR1*, and *C1QL3* using protein-coding genes only. **Table 4** presents the candidate biomarker genes that were identified from the transcriptomics experiments using Biosigner, in which three genes were differentially expressed (two downregulated and one upregulated). Three genes were identified from the models using all genes in which one of them is protein-coding, and four genes were identified from the models using the protein-coding genes. One gene (*NOTCH3*) was found common between the two groups of results (all vs. protein-coding genes). *NOTCH3* was present in all the models, and *SNAPC2* and *RP4-782L23.2* were present in two models using Biosigner. Unlike the decision tree experiments, the results were opposite in Biosigner; the models using protein-coding genes performed either similar or better results than the models using all genes. We also compared the gene list found between the decision tree models and the Biosigner models; *NOTCH3* and *SNAPC2* were found common between those two sets of genes. Both of these genes are protein-coding and were found downregulated in the differential analysis.

**Table 4:** Candidate biomarker genes from transcriptomics analysis.

Experiment name	Gene names (Experiments using all genes)	Gene names (experiments using the protein-coding genes only)
TMM + Decision Tree	*NOTCH3, TMEM106B*	*NOTCH3, TMEM106B*
qNorm + Decision Tree	*NOTCH3, RP11-792A8.1*	*NOTCH3, SMAP2*
vNorm + Decision Tree	*NOTCH3, RP11-459F6.3*	*NOTCH3, DDB2*
**TMM + GLM + Decision Tree**	***NOTCH3, KLF2P1, MFAP2***	***NOTCH3, B4GALNT1, ZNF865***
qNorm + GLM + Decision Tree	*NOTCH3, KLF2P1*	*NOTCH3, PTOV1, GTF3C5*
vNorm + GLM + Decision Tree	*NOTCH3, KLF2P1*	*NOTCH3, SNAPC2*
Biosigner (PLSDA)	*NOTCH3, RP4-782L23.2*	*NOTCH3, SNAPC2*
Biosigner (Random Forest)	*NOTCH3, RP4-782L23.2, SEMA3B-AS1*	*NOTCH3*
Biosigner (SVM)	No signature or A-tier genes were found in the final model	*NOTCH3, SNAPC2, ILDR1, C1QL3*

### Performance Evaluation of Models Using Transcriptomics Data

The results of the decision tree performance measures on the transcriptomics dataset using all genes are presented in **Table 5**. We observed that the models using all genes performed better than the models using protein-coding genes. This is mainly because, in case of all genes, the models took the benefit of using genes that are not protein-coding. When the decision tree was created on three different normalized data (TMM, qNorm, and vNorm), the model tends to perform better on the TMM data. The “TMM + Decision Tree” experiment achieved the accuracy of 71.1%, with sensitivity of 68.8%, specificity of 72.7%, and precision of 64.7%. The F_1_ score of the “TMM + Decision Tree” experiment is 0.677, MCC is 0.412, and AUC is 0.665. We also applied the GLM technique on the 14,154 genes and identified 28 DEGs: five genes were upregulated, and 23 genes were down regulated. When the decision tree was created using those 28 DEGs, the performance measures improved significantly on all three different normalized data (TMM, qNorm, and vNorm). Among these experiments, the “TMM + GLM + Decision Tree” experiment achieved the best performance followed by the “qNorm + GLM + Decision Tree” experiment that achieved the second best performance. The “TMM + GLM + Decision Tree” experiment achieved the accuracy of 89.5%, with sensitivity of 81.3%, specificity of 95.5%, and precision of 92.9%. The F_1_ score of the “TMM + GLM + Decision Tree” experiment is 0.867, MCC is 0.785, and AUC is 0.92. **Table 3** presents the decision tree model that was created using the “TMM + GLM + Decision Tree” experiment.

**Table 5:** Performance measures using transcriptomics data by leave-one-out cross-validation.

Gene feature set	Experiment name	Accuracy	Sensitivity	Specificity	Precision	F_1_ score	MCC	AUC
All	TMM + Decision Tree	0.711	0.688	0.727	0.647	0.667	0.412	0.665
All	qNorm + Decision Tree	0.184	0.000	0.318	0.000	NA	−0.689	0.239
All	vNorm + Decision Tree	0.553	0.188	0.818	0.429	0.261	0.007	0.205
All	**TMM + GLM + Decision Tree**	**0.895**	**0.813**	**0.955**	**0.929**	**0.867**	**0.785**	**0.920**
All	qNorm + GLM + Decision Tree	0.842	0.750	0.909	0.857	0.800	0.675	0.820
All	vNorm + GLM + Decision Tree	0.684	0.375	0.909	0.750	0.500	0.344	0.810
All	**Biosigner (PLSDA)**	**0.737**	**0.864**	**0.563**	**0.731**	**0.792**	**0.453**	**NA**
All	Biosigner (Random Forest)	0.447	0.455	0.438	0.526	0.488	-0.107	NA
All	Biosigner (SVM)	0.553	0.636	0.438	0.609	0.622	0.075	NA
Protein-Coding	TMM + Decision Tree	0.711	0.625	0.773	0.667	0.645	0.402	0.611
Protein-Coding	qNorm + Decision Tree	0.421	0.125	0.636	0.200	0.154	−0.268	0.554
Protein-Coding	vNorm + Decision Tree	0.263	0.125	0.364	0.125	0.125	−0.511	0.239
Protein-Coding	**TMM + GLM + Decision Tree**	**0.842**	**0.625**	**1.000**	**1.000**	**0.769**	**0.701**	**0.625**
Protein-Coding	qNorm + GLM + Decision Tree	0.763	0.563	0.909	0.818	0.667	0.513	0.577
Protein-Coding	vNorm + GLM + Decision Tree	0.763	0.563	0.909	0.818	0.667	0.513	0.573
Protein-Coding	**Biosigner (PLSDA)**	**0.763**	**0.955**	**0.500**	**0.724**	**0.824**	**0.528**	**NA**
Protein-Coding	Biosigner (Random Forest)	0.447	0.500	0.375	0.524	0.512	−0.124	NA
Protein-Coding	Biosigner (SVM)	0.605	0.591	0.625	0.684	0.634	0.213	NA

The best model with corresponding performance measures in each subgroup of experiment is presented in bold text.

For the experiments using only the protein-coding genes, the decision tree model tends to perform better on the TMM data. This is consistent with the experiments using all genes. The “TMM + Decision Tree” experiment achieved the accuracy of 71.1%, with sensitivity of 62.5%, specificity of 77.3%, and precision of 66.7%. The F_1_ score of the “TMM + Decision Tree” experiment is 0.645, MCC is 0.402, and AUC is 0.611. We also applied the GLM technique on the 11,687 protein-coding genes and identified 11 protein-coding genes that were differentially expressed in which one gene was upregulated and 10 genes were downregulated. When the decision tree was created using those 11 protein-coding DEGs, the performance measures improved significantly on all three different normalized data (TMM, qNorm, and vNorm). This is also consistent with the experiments using all genes. Among these experiments, the “TMM + GLM + Decision Tree” experiment achieved the best performance, which is 84.2% accuracy, 62.5% sensitivity, 100% specificity, and 100% precision. The F_1_ score of the “TMM + GLM + Decision Tree” experiment is 0.769, MCC is 0.701, and AUC is 0.625. **Table 3** presents the decision tree model that was created using the “TMM + GLM + Decision Tree” experiment; the model shows the gene names that were identified in the decision tree models differentiating endometriosis vs. control.

**Table 5** presents the Biosigner performance measures that were computed using the leave-one-out cross-validation approach. For both groups of results (all vs. protein-coding genes), the best performance was observed using the PLSDA model. For all genes, the PLSDA achieved the accuracy of 73.7%, sensitivity of 86.4%, specificity of 56.3%, precision of 73.1%, F_1_ score of 0.792%, and MCC of 0.453. For the protein-coding genes, the PLSDA achieved the accuracy of 76.3%, sensitivity of 95.5%, specificity of 50.0%, precision of 72.4%, F_1_ score of 0.824, and MCC of 0.528. The performance measures for random forest and SVM are significantly lower in comparison with those for PLSDA using all genes. However, SVM has a higher specificity than PLSDA and random forest using protein-coding genes.

**Figure 3:**
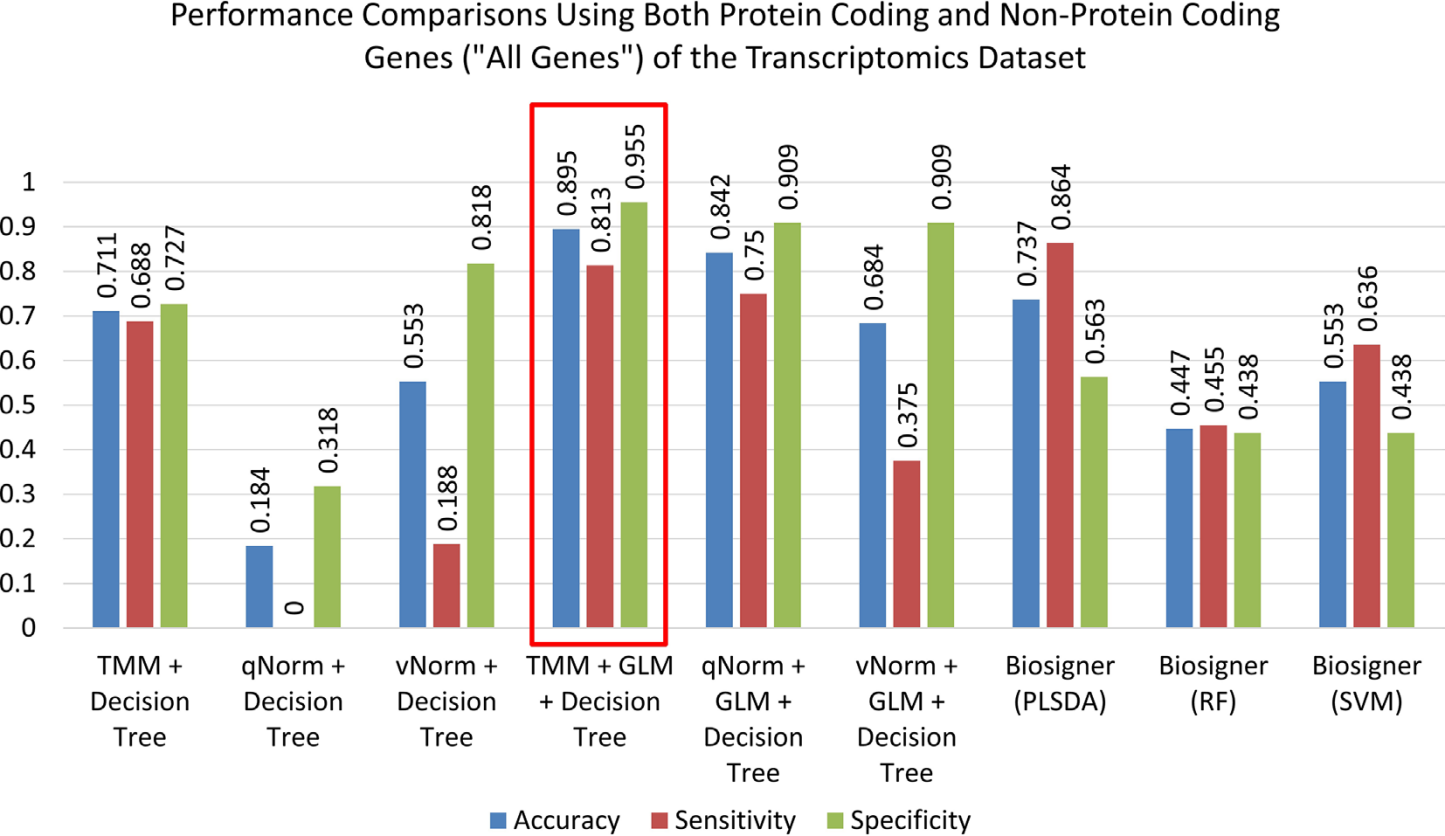
Performance comparisons using both protein-coding and nonprotein-coding genes (“all genes”) of the transcriptomics dataset.

**Figure 4:**
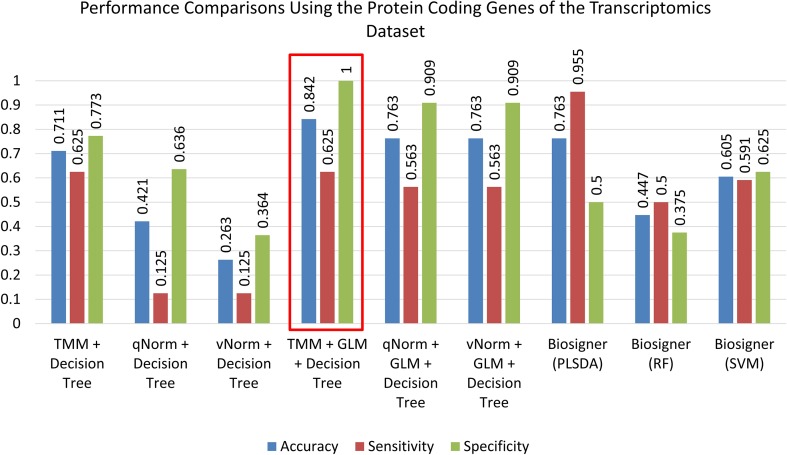
Performance comparisons using the protein-coding genes of the transcriptomics dataset.

A bar chart comparison of accuracy, sensitivity, and specificity for experiments using all genes is presented in **Figure 3**. In this scenario, the “TMM + Decision Tree” experiment has a balanced accuracy, sensitivity, and specificity but does not outperform all of the experiments. The “TMM + GLM + Decision Tree” experiment produced the highest accuracy, specificity, and precision among all the experiments and outperformed all of the experiments by F_1_ score and MCC. A bar chart comparison of accuracy, sensitivity, and specificity for experiments using the protein-coding genes is presented in **Figure 4**. In this scenario, the SVM in Biosigner has a balanced accuracy, sensitivity, and specificity but does not outperform all of the experiments. The PLSDA in Biosigner achieved the best sensitivity and F_1_ score but has a poor specificity. The “TMM + GLM + Decision Tree” experiment produced the highest accuracy, specificity, and precision among all of the experiments and outperformed all experiments based on MCC. In both scenarios (all vs. protein-coding genes), GLM was useful for improving the overall performance in case of the decision tree application.

### Decision Tree Results Using Methylomics Data

**Figure 5:**
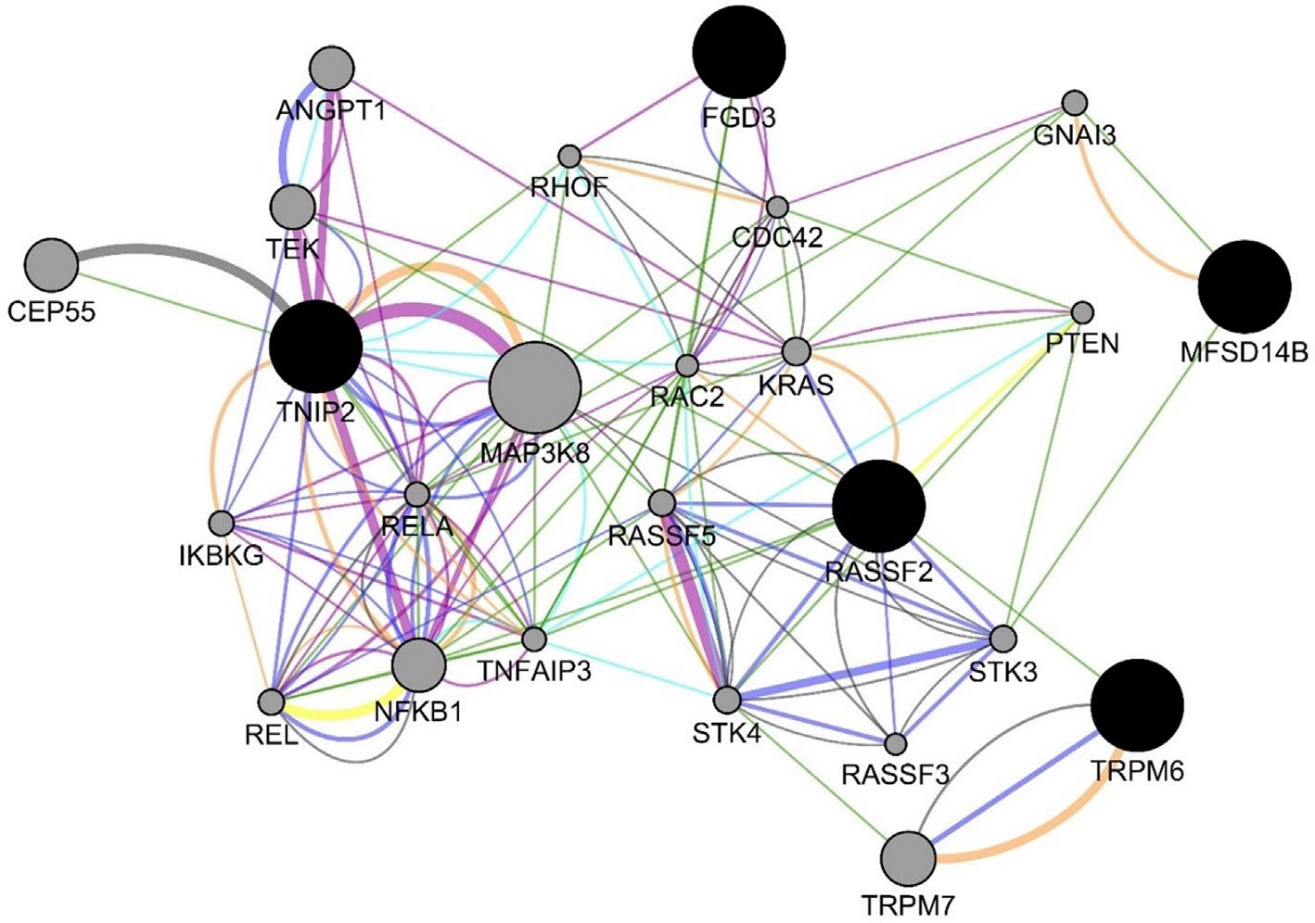
Gene interaction network among the genes from the decision tree models using the methylomics data. The blue, green, orange, teal, purple, yellow, and gray edges represent various GeneMANIA networks: physical interaction network (weight 67.64), co-expression network (weight 13.50), predicted functional relationships between genes (weight 6.35), co-localization network (6.17), pathway network (weight 4.35), genetic interaction network (weight 1.40), and shared protein domain network (weight 0.59), respectively.

For the six decision tree experiments using the methylomics dataset, we used 2,577,382 methylated regions in these experiments. **Table 6** presents the decision tree models from different experiments. The methylated regions of interest (MROI) were extracted from the decision tree models, and the nearby genes of those MROIs were extracted using the process described in the Methods section. All the MROIs and nearby genes are presented in **Table 7**. Among those 17 MROIs, eight regions were differentially methylated and hypo-methylated. We found eight nearby genes of those 17 MROIs within the distance of 10,000 bp, in which five genes are protein-coding (e.g., *MFSD14B*, *RASSF2*, *TRPM6*, *TNIP2*, and *FGD3*), two genes are lincRNA (e.g., *RP11-734K21.4* and *RP3-522J7.6*), and one is pseudogene (e.g., *RPL37AP1*). Also, the MROIs related to five genes (e.g., *MFSD14B*, *RASSF2*, *RP11-734K21.4*, *RP3-522J7.6*, and *TNIP2*) were found upstream and three genes (e.g., *RPL37AP1*, *TRPM6*, and *FGD3*) overlapped with the regions. **Figure 5** presents the gene–gene interaction network comprising all the nearby genes of the MROIs identified by the decision tree models. GeneMANIA predicted many genes that are closely related to the query genes. The gene interaction network includes physical interaction network (weight 67.64), co-expression network (weight 13.50), predicted functional relationships between genes (weight 6.35), co-localization network (6.17), pathway network (weight 4.35), genetic interaction network (weight 1.40), and shared protein domain network (weight 0.59). Some of the top gene ontologies from the functional enrichment analysis include regulation of endothelial cell apoptotic process, toll-like receptor signaling pathway, innate immune response-activating signal transduction, Fc receptor signaling pathway, and regulation of I-kappaB kinase/NF-kappaB signaling. More detail is presented in the **Supplementary Table 1**.

**Table 6:** Decision tree models using methylomics data.

Experiment Name	Tree Model
TMM + Decision Tree	chr2_147728001_147729000 <= 1.207401::...chr10_132354001_132355000 <= 1.41709: endometriosis (2): chr10_132354001_132355000 > 1.41709: control (22)chr2_147728001_147729000 > 1.207401::...chr1_35106001_35107000 <= 1.102261: :...chr1_20862001_20863000 <= 0.1675461: endometriosis (2) : chr1_20862001_20863000 > 0.1675461: control (11) chr1_35106001_35107000 > 1.102261: :...chr22_16562001_16563000 <= 0.286556: control (2) chr22_16562001_16563000 > 0.286556: endometriosis (38)
qNorm + Decision Tree	chr9_94372001_94373000 > 5.356569::...chr1_3182001_3183000 <= 5.492207: endometriosis (2): chr1_3182001_3183000 > 5.492207: control (22)chr9_94372001_94373000 <= 5.356569::...chr1_2908001_2909000 > 5.999371: control (7) chr1_2908001_2909000 <= 5.999371: :...chr16_37922001_37923000 <= 4.049325: control (5) chr16_37922001_37923000 > 4.049325: endometriosis (41/1)
vNorm + Decision Tree	chr9_94372001_94373000 > 1.435922::...chr1_3182001_3183000 <= 1.56803: endometriosis (2): chr1_3182001_3183000 > 1.56803: control (22)chr9_94372001_94373000 <= 1.435922::...chr1_2908001_2909000 > 2.063281: control (7) chr1_2908001_2909000 <= 2.063281: :...chr16_37922001_37923000 <= 0.1332516: control (5) chr16_37922001_37923000 > 0.1332516: endometriosis (41/1)
TMM + GLM + Decision Tree	chr9_92948001_92949000 > 0.1864191: control (19/1)chr9_92948001_92949000 <= 0.1864191::...chr2_9142001_9143000 > 0.515642: control (8) chr2_9142001_9143000 <= 0.515642: :...chr4_2757001_2758000 > 0.9228122: control (5) chr4_2757001_2758000 <= 0.9228122: :...chr22_49841001_49842000 <= 1.252199: endometriosis (41/1) chr22_49841001_49842000 > 1.252199: control (4/1)
**qNorm + GLM + Decision Tree**	chr9_74884001_74885000 <= 4.534801::...chr20_4827001_4828000 <= 4.961341: endometriosis (30): chr20_4827001_4828000 > 4.961341: control (5/1)chr9_74884001_74885000 > 4.534801::...chr10_71353001_71354000 > 4.124296: control (29/1) chr10_71353001_71354000 <= 4.124296: :...chr20_44466001_44467000 <= 5.021993: endometriosis (10) chr20_44466001_44467000 > 5.021993: control (3)
**vNorm + GLM + Decision Tree**	chr9_74884001_74885000 <= 0.280641::...chr20_4827001_4828000 <= 0.7003891: endometriosis (30): chr20_4827001_4828000 > 0.7003891: control (5/1)chr9_74884001_74885000 > 0.280641::...chr10_71353001_71354000 > -0.1261655: control (29/1) chr10_71353001_71354000 <= -0.1261655: :...chr20_44466001_44467000 <= 0.7601537: endometriosis (10) chr20_44466001_44467000 > 0.7601537: control (3)

**Table 7:** Methylated regions of interest (MROI) and candidate biomarker genes from methylomics analysis.

Experiment name	Methylated regions of interest (MROI)	Nearby gene names
TMM + Decision Tree	chr2_147728001_147729000, chr10_132354001_13235500, chr1_35106001_35107000, chr1_20862001_20863000, chr22_16562001_16563000	Not found
qNorm + Decision Tree	chr9_94372001_94373000, chr1_3182001_3183000, chr1_2908001_2909000, chr16_37922001_37923000	*MFSD14B*
vNorm + Decision Tree	chr9_94372001_94373000, chr1_3182001_3183000, chr1_2908001_2909000, chr16_37922001_37923000	*MFSD14B*
TMM + GLM + Decision Tree	chr9_92948001_92949000, chr2_9142001_9143000, chr4_2757001_2758000, chr22_49841001_49842000	*RP11-734K21.4, RP3-522J7.6, TNIP2, FGD3*
**qNorm + GLM + Decision Tree**	**chr9_74884001_74885000, chr20_4827001_4828000, chr10_71353001_71354000, chr20_44466001_44467000**	***RPL37AP1, RASSF2, TRPM6***
**vNorm + GLM + Decision Tree**	**chr9_74884001_74885000, chr20_4827001_4828000, chr10_71353001_71354000, chr20_44466001_44467000**	***RPL37AP1, RASSF2, TRPM6***
Biosigner (PLSDA)	chr7_5111001_5112000, chr5_29429001_29430000, chr22_49841001_49842000	*RP3-522J7.6*, *OR10AH1P*
Biosigner (Random Forest)	chr11_2027001_2028000, chr2_147728001_147729000, chr9_74884001_74885000	*TRPM6*
Biosigner (SVM)	chr18_17526001_17527000, chr4_186970001_186971000, chr4_189277001_189278000	Not found

The best model is presented in bold text.

### Biosigner Results Using Methylomics Data

**Figure 6:**
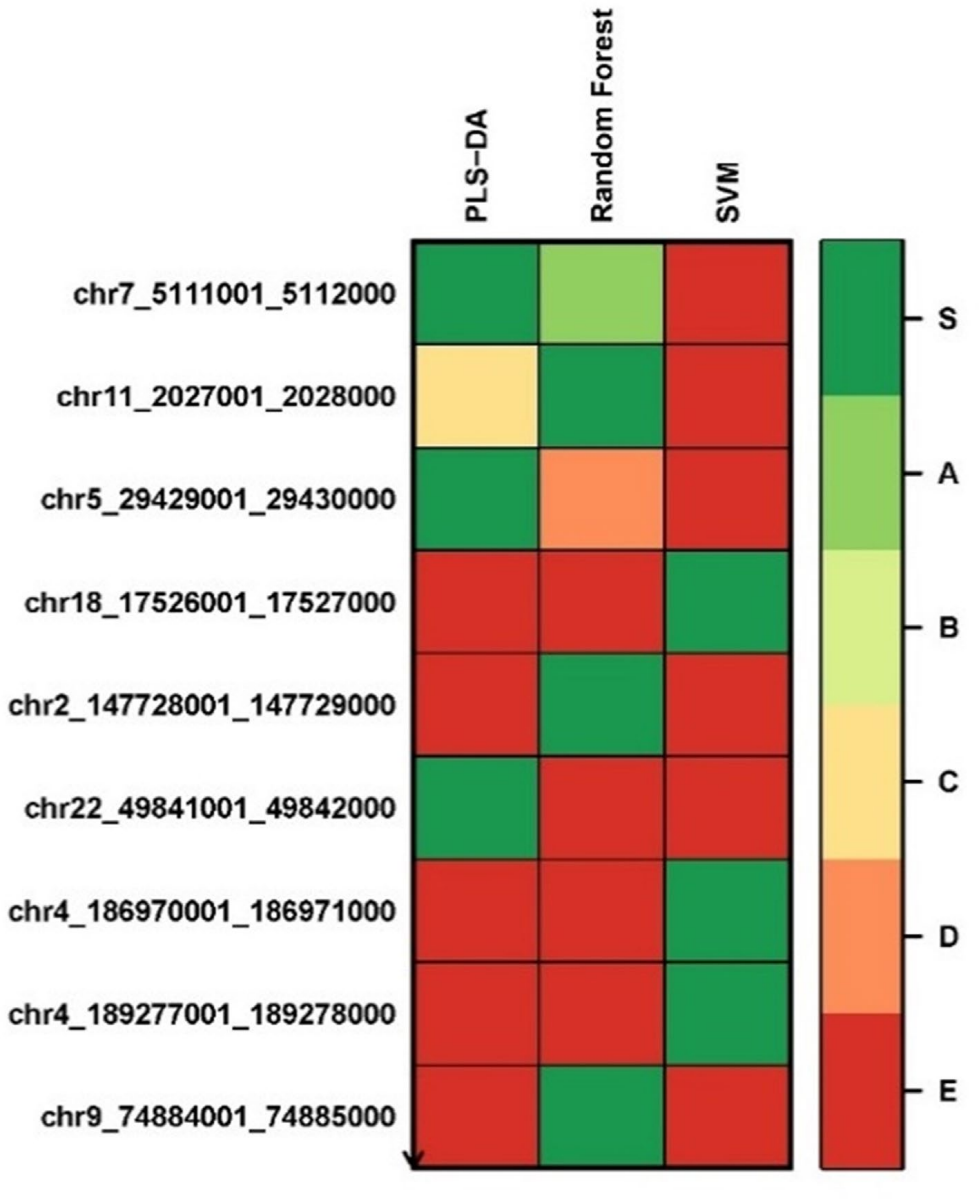
Methylated region tier plot from biosigner using methylomics data; S, signature genes; A–E=A is a higher tier, and E is a lower tier.

We applied the Biosigner algorithm on 2,577,382 methylated regions. As the input of the Biosigner algorithm, we used the TMM normalized data as the methylation expression data. The Biosigner algorithm constructed three different models: PLSDA, random forest, and SVM. **Figure 6** presents the tier plot of the methylated regions (here, S = signature genes; A–E = A is a higher tier gene and E is a lower tier genes). Biosigner identified nine methylated regions as the potential set of biomarkers. The MROIs (*n = 9*) were extracted from the Biosigner models, and the nearby genes of those MROIs were extracted using the experiments described in the *Methods* section. Among those nine MROIs, four regions were differentially methylated (three hypo-methylated and one hyper-methylated). We found three genes (see **Table 7**) within the distance of 10,000 bp from those nine MROIs, in which one gene is protein-coding (e.g., *TRPM6*), one is lincRNA (e.g., *RP3-522J7.6*), and one is pseudogene (e.g., *OR10AH1P*). Also, the MROIs related to two genes (e.g., *RP3-522J7.6* and *OR10AH1P*) were found upstream and one (e.g., *TRPM6*) overlapped with the regions. We compared the genes found from the decision tree and the Biosigner experiments and found two common genes (e.g., *TRPM6* and *RP3-522J7.6*).

### Performance Evaluation of Models Using Methylomics Data

The experimental results of the decision tree performance measures on the methylomics dataset are presented in **Table 8**. When the decision tree was created on three different normalized data (TMM, qNorm, and vNorm), the model tends to perform better on TMM normalization. The “TMM + Decision Tree” experiment achieved the accuracy of 40.3%, with sensitivity of 52.4%, specificity of 25.7%, and precision of 45.8%. The F_1_ score of the “TMM + Decision Tree” experiment is 0.489, MCC is −0.225, and AUC is 0.414. We also applied the differential analysis using GLM on the 2,577,382 methylated regions and identified 365 DMRs. When the decision tree was created using those 365 DMRs, the performance measures improved significantly on all three different normalized data. Among these experiments, both “qNorm + GLM + Decision Tree” and “vNorm + GLM + Decision Tree” experiments achieved the best performance. These experiments achieved the accuracy of 77.9%, with sensitivity of 76.2%, specificity of 80.0%, and precision of 82.1%. The F_1_ score of these experiments is 0.790, MCC is 0.560, and AUC is 0.721.

**Table 8:** Performance measures using methylomics data by leave-one-out cross-validation.

Experiment name	Accuracy	Sensitivity	Specificity	Precision	F_1_ score	MCC	AUC
**TMM + Decision Tree**	**0.403**	**0.524**	**0.257**	**0.458**	**0.489**	**−0.225**	**0.414**
qNorm + Decision Tree	0.364	0.405	0.314	0.415	0.410	−0.280	0.199
vNorm + Decision Tree	0.403	0.405	0.400	0.447	0.425	−0.194	0.233
TMM + GLM + Decision Tree	0.714	0.714	0.714	0.750	0.732	0.427	0.679
**qNorm + GLM + Decision Tree**	**0.779**	**0.762**	**0.800**	**0.821**	**0.790**	**0.560**	**0.721**
**vNorm + GLM + Decision Tree**	**0.779**	**0.762**	**0.800**	**0.821**	**0.790**	**0.560**	**0.721**
**Biosigner (PLSDA)**	**0.688**	**0.600**	**0.762**	**0.677**	**0.636**	**0.367**	**NA**
Biosigner (Random Forest)	0.429	0.314	0.524	0.355	0.333	−0.164	**NA**
Biosigner (SVM)	0.519	0.400	0.619	0.467	0.431	0.019	**NA**

The best model with corresponding performance measures in each subgroup of experiment is presented in bold text.

**Table 8** presents the Biosigner performance measures that were computed using the leave-one-out cross-validation approach. The best performance was observed using the PLSDA model, with accuracy of 68.8%, sensitivity of 60.0%, specificity of 76.2%, precision of 67.7%, F_1_ score of 0.636%, and MCC of 0.367. The performance measures for random forest and SVM are significantly lower in comparison with those for PLSDA.

**Figure 7:**
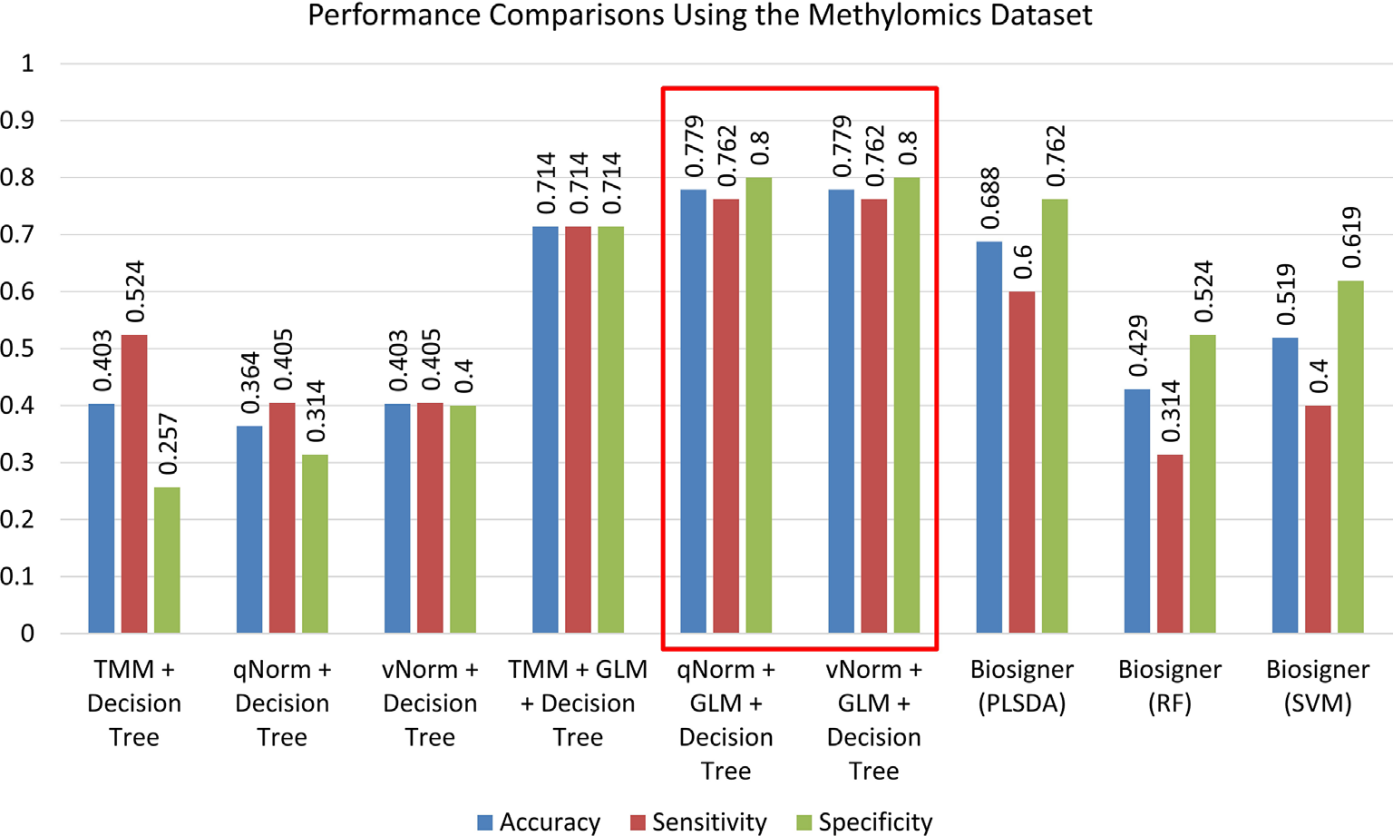
Performance comparisons using the methylomics dataset.

A bar chart comparison of accuracy, sensitivity, and specificity for experiments using the methylomics dataset is presented in **Figure 7**. The “qNorm + GLM + Decision Tree” and “vNorm + GLM + Decision Tree” experiments have a balanced accuracy, sensitivity, and specificity and outperform all of the experiments. Both the “qNorm + GLM + Decision Tree” and “vNorm + GLM + Decision Tree” experiments produced the highest accuracy, sensitivity, and specificity among all the experiments and outperformed all of the experiments by F_1_ score, and MCC. GLM was useful for improving the overall performance in case of decision tree application.

## Discussion

This work achieves the aim of broadly assessing how well the supervised machine learning classifiers perform in classifying endometriosis vs. control samples using the whole genome next generation transcriptomics data and methylomics data as well as facilitate identifying candidate biomarker genes. Since the machine learning training process is a data-driven approach, we wanted to assess multiple aspects by various experiments and several important conclusions were made.

First, we evaluated three different normalization techniques. We found that the performance of machine learning classifiers varied depending on the normalization techniques, but the choice of normalization should be based on the type of dataset. For the endometriosis classification task, our experiment revealed that TMM normalization performed the best for the transcriptomics dataset, and both qNorm and vNorm performed the best for the methylomics dataset. This finding is consistent with the results demonstrated by the study on comparing normalization techniques using transcriptomics data (Lin et al., 2016).

Second, the differential analysis using the GLM is an established method for identifying the DEGs from the transcriptomics datasets and for identifying the DMRs from the methylomics datasets. We used GLM in combination with decision tree. We found that GLM was useful for improving the performance of decision tree models.

Third, candidate biomarker genes can be extracted from the machine learning models. From the transcriptomics analysis, *NOTCH3* has been identified as a candidate biomarker by all of the methods, which is a protein-coding gene and found to be differentially expressed and downregulated. In all the variations of the decision tree experiments in this work, *NOTCH3* was chosen as the primary differentiating criteria (root node). Recent studies recommend that *NOTCH3* signaling may play a major role in oncogenesis, tumor maintenance, and resistance to chemotherapy (Aburjania et al., 2018). Prior study also reported that dysregulation and decrease in *NOTCH* signaling pathway are associated with endometriosis (Su et al., 2015; González-Foruria et al., 2017). *NOTCH3* has also been reported as a major driver for breast cancer development (Braune et al., 2018). It plays an important role in maintaining the tumor phenotype in pancreatic ductal adenocarcinoma (PDAC) (Song et al., 2018), lung carcinogenesis (Su et al., 2018), and endometrial carcinoma (Mitsuhashi et al., 2012). Also, from the transcriptomics data analysis, *SNAPC2* was identified by the decision tree and Biosigner experiments. A recent genome-wide methylation study proposes *SNAPC2* as a biomarker for glioblastoma prediction (Ma et al., 2015). Other candidate biomarker genes are also reported to be associated with endometriosis and/or different types of cancers. Another study identified *B4GALNT1* to be related to endometrial cancer (Trimarchi et al., 2017). *GTF3C5* has been reported as differently expressed between endometrioid endometrial cancer and non-endometrioid endometrial cancer. *TMEM106B* was found upregulated in ectopic versus eutopic endometrium of women with endometriosis (Meola et al., 2010). *MFAP2* stimulates epithelial–mesenchymal transition in gastric cancer cells by activating *TGFβ signaling pathway* that supports survival and metastasis of endometrial cancer cells (Lei et al., 2009). *MFAP2* is also related to human endometrial receptivity (Díaz-Gimeno et al., 2011) and has been defined as biochemical pregnancy biomarkers (Fung et al., 2018). Other study found *MFAP2* as differentially expressed in severe vs. mild endometriosis (Fung et al., 2018). *SMAP2* was reported to be involved in microsatellite instability oncogenesis (Sangar et al., 2014). A recent study reported that overexpressed *PTOV1* plays a major role in tumorigenesis and progression of esophageal cancer (Li et al., 2017) and in prostate cancer (Benedit et al., 2001). *ZNF865* coordinates the functionality of cancer networks (Ghanat Bari et al., 2017). *DDB2* enhances tumorigenesis and different types of cancers (Romieu-Mourez et al., 2001; Barakat et al., 2010; Meola et al., 2010; Han et al., 2014). *ILDR1* was identified as a diagnostic marker for cancer progression (Hauge et al., 2004). Further details of these genes are provided in the **Supplementary Table 2**.

From the methylomics analysis, two genes (e.g., *TRPM6* and *RP3-522J7.6*) were identified by both the decision tree and Biosigner experiments. *TRPM6* is known to be related to two pathways (e.g., *CREB Pathway* and *Ion channel transport*) and associated with a disease named *Hypomagnesemia*. GO annotations of *TRPM6* include protein serine/threonine kinase activity and calcium channel activity. Serine/threonine kinase activity has been reported to be associated with endometriosis (Kao et al., 2003). *RP3-522J7.6* is a lincRNA. Methylomics analysis also revealed some other clinically significant genes. *RASSF2* is a tumor suppressor gene and was proposed as a novel methylation marker for screening several cancers (Cooper et al., 2008). *RASSF2* has been reported to be associated with ovarian endometriosis (Ren et al., 2014). The GO annotations related to *TNIP2* includes protein kinase binding and polyubiquitin modification-dependent protein binding. *TNIP2* is a hub protein in the NF-κB network (Banks et al., 2016), and NF-kB has an important role in the pathophysiology of endometriosis (Kaponis et al., 2012). *FGD3* was reported to be associated with six distinct breast cancer cohorts and four TCGA cancer cohorts and was proposed as an important clinical biomarker for cancers (Willis et al., 2017). *MFSD14B* is known as neuronal and affected by nutrient availability (Lekholm et al., 2017), and GO annotations include transporter activity. Three of the RAS-association domain family members (*RASSF2*, *RASSF3*, and *RASSF5*) were involved in the network analysis (**Figure 5**) using all methylomics decision tree models, which are known as tumor suppressor genes and epigenetically inactivated in different tumor types. *RASSF2* was reported to be associated with ovarian endometriosis, breast cancer, gastric cancer, and childhood acute lymphoblastic leukemia (Ren et al., 2014; Perez-Janices et al., 2015; Aydin et al., 2016; Singh et al., 2016) and has been proposed as a novel methylation marker for screening several cancers (Cooper et al., 2008). *RASSF3* is an oncogene and mutated in nearly one third of all human cancers. Somatic mutations and other genomic abnormalities were also found in patients with endometriosis that are associated in cancer development. *PTEN* is a tumor suppressor gene and mostly occurs in endometrial and ovarian cancers. Somatic mutations in the *PTEN* gene were identified in 20% ovarian endometrioid carcinomas, 8.3% clear cell carcinomas, and 20.6% solitary endometrial cysts (Sato et al., 2000). *KRAS* plays a role in promoting oncogenic events in colorectal cancer. Mutations in the *KRAS* gene were found in patients with endometriosis (Vestergaard et al., 2011; Anglesio et al., 2017). *MAP3K8* activation is critically involved in both inflammation and oncogenetic events (Vougioukalaki et al., 2011; Lee et al., 2015). *MAP3K8* was identified as an oncogene in endometrial cancer, breast cancer, colon cancer, renal cancer, gastric cancer, and nasopharyngeal carcinoma (Lee et al., 2015), but it is a tumor suppressor gene in lung and intestinal cancers (Gkirtzimanaki et al., 2013; Zhang et al., 2016) as well. *MAP3K8* was found upregulated in multiple tumor types and closely related to tumorigenesis (Sperger et al., 2003; D’Errico et al., 2009).

The findings of many cancer-associated genes in our study were surprising but not new. Though endometriosis is considered to be a benign condition, some of the characteristics of endometriosis are similar to cancer; for example, both endometriosis and cancer can be metastatic, angiogenic, and resistant to apoptosis. In the past, several studies examined if endometriosis has any relation with cancer. Recent studies have found cancer-associated mutations in endometriotic lesions (Sato et al., 2000; Thomas and Campbell, 2000) and also in deep infiltrating endometriosis without coexisting cancer (Anglesio et al., 2017). Other studies have shown that endometrial cancer and endometriosis (both are estrogen dependent and a disease of chronic inflammation) appear to have a moderate but significant shared genetic correlation (Wenzl et al., 2003; Painter et al., 2018). A recent study, based on The National Health Insurance Research Databases in Taiwan, has claimed that there is a potential association between endometriosis and endometrial cancer. This study has reported that the endometriosis patients have higher risk for developing endometrial cancer in their later life, with an adjusted hazard ratio (aHR) of 2.83 [95% confidence interval (CI) = 1.49 to 5.35], and for older women (age >40) diagnosed with endometriosis, the ratio was higher (aHR = 7.08, 95% CI = 2.33 to 21.55) (Yu et al., 2015). Another study has identified that 85% of atypical endometriosis lesions have a cancer-like immunological gene signature (Edwards et al., 2015). It has been reported that endometriosis is associated with ovarian cancer (Jimbo et al., 1997) and has a fourfold increased risk of developing the ovarian cancer (Kok et al., 2015). Also, there is a shared genetic risks between endometriosis and epithelial ovarian cancer (Lu et al., 2015). There is a significant risk of developing breast cancer in patients with endometriosis (Schairer et al., 1997; Chuang et al., 2015). The overall cancer risk has been found higher, with a standardized incidence ratio (SIR) of 1.2 (95% CI 1.1 to 1.3) in a study on 20,686 endometriosis patients who were hospitalized during the period 1969 to 1983 in Sweden. The SIR was 1.3 (95% CI 1.1 to 1.4) for breast cancer, 1.9 for ovarian cancer (95% CI 1.3 to 2.8), and 1.4 (95% CI 1.0 to 1.8) for hematopoietic malignancies (Brinton et al., 1997). Another study, based on 63,630 women with endometriosis, has found that endometriosis patients has an increased risk for several malignancies. The SIR for endocrine tumors was 1.38, ovarian cancer was 1.37, renal cancer was 1.36, thyroid cancer was 1.33, brain tumors was 1.27, malignant melanoma was 1.23, and breast cancer was 1.08 (Melin et al., 2007). Endometriosis has been found to be associated with an increased overall risk of skin cancer, with a hazard ratio (HR) of 1.28 (95% CI 1.05 to 1.55) and melanoma risk with HR 1.64 (95% CI 1.15 to 2.35) (Farland et al., 2017).

Fifth, machine learning classifiers can be trained for creating highly accurate models for classifying endometriosis with high sensitivity and specificity thus creating the opportunity for precision medicine application for endometriosis. The diagnostic latency of endometriosis is very high, with an average delay of 4–11 years mainly because of complexity in diagnosis techniques. The machine learning models in this study achieved a high F_1_ score (0.867) for the transcriptomics dataset and a high F_1_ score (0.79) for the methylomics dataset. The current diagnostic process is highly invasive in nature, but we anticipate a future where a minimally invasive endometrial biopsy with a machine learning predictive diagnostic model as demonstrated in this study can be used for diagnosing endometriosis. In recent years, we have seen the success of deep learning in various domains including bioinformatics such as described in a review article (Li et al., 2019). Another study demonstrated the application of deep learning for mutation map analysis (Umarov et al., 2019). A future extension of this study could be to apply the deep learning techniques for classifying endometriosis and demonstrate a process for interpretation of the models for biomarker detection.

Finally, we found that the type of whole genome sequencing data has an impact on the predictive performance. Transcriptomic dataset achieved higher accuracy in comparison with the methylomics dataset. An interesting investigation would be to develop an integrative classification model by integrating both transcriptomes and methylomics data to train a single model and measure the predictive performance. This aim is supported by our rationale that an integrative multi-omics approach may increase predictive performance thus providing a highly accurate predictive diagnostic model. Further study is needed for investigating such hypothesis.

In summary, this study demonstrated that a supervised machine learning method leveraging transcriptomics or methylomics data is a reliable approach for classifying endometriosis. We concluded that an appropriate machine learning diagnostic pipeline for endometriosis should use a) either transcriptomics or methylomics data, b) TMM normalization for the transcriptomics data, or qNorm or vNorm for the methylomics data, and c) GLM for feature space reduction and classification performance maximization. The conclusion was made based on the use case of endometriosis classification in this study. Further study is needed to generalize the results across multiple disease classification cases as well as using publicly available data on multiple populations.

## Data Availability

The datasets GENERATED for this study can be found in the gene Expression Omnibus, https://www.ncbi.nlm.nih.gov/geo/query/acc.cgi?acc=GSE134052; https://www.ncbi.nlm.nih.gov/geo/query/acc.cgi?acc=GSE134056

## Ethics Statement

The protocol was approved by the University of Missouri Institutional Review Board. The physician obtained informed consent following the IRB protocol. All subjects gave informed consent in accordance with the Declaration of Helsinki.

## Author Contributions

SA, TJ, DX, and SN contributed in the conception and design of the study; SN, TJ, JB, KP, and GW contributed in the acquisition of the samples and data. SA organized and preprocessed the data, performed the machine learning analysis, and wrote the first draft of the manuscript. All authors contributed to the manuscript revision and read and approved the submitted version.

## Funding

This work was supported by NIEHS R21 ES020039, University of Missouri Institute for Clinical and Translational Science, NIH/National Center for Advancing Translational Sciences (NCATS) grant UL1TR002345, and University of Missouri Research Council Grant.

## Conflict of Interest Statement

The authors declare that the research was conducted in the absence of any commercial or financial relationships that could be construed as a potential conflict of interest.
