# Supplementary material for: Machine Learning Classifiers for Endometriosis Using Transcriptomics and Methylomics Data
Source: Front Genet. 2019 Sep 4;10:766. doi: 10.3389/fgene.2019.00766 (PMC6737999; doi:10.3389/fgene.2019.00766)
Supplement: Supplementary file 1 [file Table_1.docx]

Supplementary Table 1: Functional Enrichment Analysis Using the Genes from the Decision Tree Models Using the Methylomics Data

| **GO ID** | **Description** | **q-value** | **Coverage ratio** |
| --- | --- | --- | --- |
| GO:2000352 | negative regulation of endothelial cell apoptotic process | 0.0000 | 4/14 |
| GO:2000351 | regulation of endothelial cell apoptotic process | 0.0001 | 4/22 |
| GO:0072577 | endothelial cell apoptotic process | 0.0002 | 4/25 |
| GO:0034134 | toll-like receptor 2 signaling pathway | 0.0002 | 5/77 |
| GO:0034138 | toll-like receptor 3 signaling pathway | 0.0002 | 5/83 |
| GO:0038093 | Fc receptor signaling pathway | 0.0009 | 6/219 |
| GO:0002224 | toll-like receptor signaling pathway | 0.0021 | 5/137 |
| GO:0002758 | innate immune response-activating signal transduction | 0.0031 | 5/160 |
| GO:0002768 | immune response-regulating cell surface receptor signaling pathway | 0.0031 | 6/296 |
| GO:0002221 | pattern recognition receptor signaling pathway | 0.0031 | 5/158 |
| GO:0038095 | Fc-epsilon receptor signaling pathway | 0.0033 | 5/166 |
| GO:0002218 | activation of innate immune response | 0.0033 | 5/168 |
| GO:0034162 | toll-like receptor 9 signaling pathway | 0.0034 | 4/74 |
| GO:0002429 | immune response-activating cell surface receptor signaling pathway | 0.0035 | 5/175 |
| GO:0043122 | regulation of I-kappaB kinase/NF-kappaB signaling | 0.0042 | 5/185 |
| GO:0045089 | positive regulation of innate immune response | 0.0047 | 5/191 |
| GO:0050852 | T cell receptor signaling pathway | 0.0051 | 4/89 |
| GO:0007249 | I-kappaB kinase/NF-kappaB signaling | 0.0051 | 5/198 |
| GO:0070423 | nucleotide-binding oligomerization domain containing signaling pathway | 0.0075 | 3/30 |
| GO:0032606 | type I interferon production | 0.0081 | 4/104 |
| GO:0032479 | regulation of type I interferon production | 0.0081 | 4/104 |
| GO:0034142 | toll-like receptor 4 signaling pathway | 0.0083 | 4/107 |
| GO:0031349 | positive regulation of defense response | 0.0083 | 5/231 |
| GO:0050851 | antigen receptor-mediated signaling pathway | 0.0098 | 4/113 |
| GO:0045088 | regulation of innate immune response | 0.0098 | 5/246 |
| GO:0071347 | cellular response to interleukin-1 | 0.0098 | 3/36 |
| GO:0002753 | cytoplasmic pattern recognition receptor signaling pathway | 0.0128 | 3/40 |
| GO:0048011 | neurotrophin TRK receptor signaling pathway | 0.0153 | 5/274 |
| GO:0038179 | neurotrophin signaling pathway | 0.0156 | 5/277 |
| GO:0006954 | inflammatory response | 0.0167 | 5/283 |
| GO:0043123 | positive regulation of I-kappaB kinase/NF-kappaB signaling | 0.0168 | 4/138 |
| GO:0035872 | nucleotide-binding domain, leucine rich repeat containing receptor signaling pathway | 0.0173 | 3/47 |
| GO:0007264 | small GTPase mediated signal transduction | 0.0173 | 5/291 |
| GO:0070555 | response to interleukin-1 | 0.0182 | 3/49 |
| GO:0060070 | canonical Wnt signaling pathway | 0.0182 | 4/145 |
| GO:0004702 | receptor signaling protein serine/threonine kinase activity | 0.0310 | 3/59 |
| GO:0043539 | protein serine/threonine kinase activator activity | 0.0370 | 2/10 |
| GO:0034146 | toll-like receptor 5 signaling pathway | 0.0383 | 3/65 |
| GO:0034166 | toll-like receptor 10 signaling pathway | 0.0383 | 3/65 |
| GO:0051091 | positive regulation of sequence-specific DNA binding transcription factor activity | 0.0384 | 4/182 |
| GO:0038124 | toll-like receptor TLR6:TLR2 signaling pathway | 0.0457 | 3/71 |
| GO:0038123 | toll-like receptor TLR1:TLR2 signaling pathway | 0.0457 | 3/71 |
| GO:0097285 | cell-type specific apoptotic process | 0.0457 | 4/194 |
| GO:0051896 | regulation of protein kinase B signaling | 0.0460 | 3/72 |
| GO:0016055 | Wnt signaling pathway | 0.0467 | 4/203 |
| GO:0010810 | regulation of cell-substrate adhesion | 0.0467 | 3/75 |
| GO:0032481 | positive regulation of type I interferon production | 0.0467 | 3/74 |
| GO:0035666 | TRIF-dependent toll-like receptor signaling pathway | 0.0467 | 3/76 |
| GO:0032147 | activation of protein kinase activity | 0.0467 | 4/202 |
| GO:0031293 | membrane protein intracellular domain proteolysis | 0.0467 | 2/13 |
| GO:0030155 | regulation of cell adhesion | 0.0467 | 4/201 |
| GO:0051249 | regulation of lymphocyte activation | 0.0495 | 4/208 |
| GO:0002756 | MyD88-independent toll-like receptor signaling pathway | 0.0504 | 3/79 |
| GO:0002755 | MyD88-dependent toll-like receptor signaling pathway | 0.0513 | 3/80 |
| GO:0030496 | midbody | 0.0523 | 3/81 |
| GO:0043491 | protein kinase B signaling | 0.0533 | 3/82 |
| GO:0002694 | regulation of leukocyte activation | 0.0686 | 4/232 |
| GO:0032102 | negative regulation of response to external stimulus | 0.0700 | 3/91 |
| GO:0043114 | regulation of vascular permeability | 0.0785 | 2/18 |
| GO:0005057 | receptor signaling protein activity | 0.0842 | 3/98 |
| GO:0050865 | regulation of cell activation | 0.0906 | 4/254 |
