# Supplementary material for: Machine Learning Classifiers for Endometriosis Using Transcriptomics and Methylomics Data
Source: Front Genet. 2019 Sep 4;10:766. doi: 10.3389/fgene.2019.00766 (PMC6737999; doi:10.3389/fgene.2019.00766)
Supplement: Supplementary file 2 [file Table_2.docx]

Supplementary Table 2: GO Annotation, Pathways and the Associated Diseases for the Genes from the Decision Tree Models Using Transcriptomics Data

| **Gene** | **GO annotation** | **Pathways** | **Diseases Associated** |
| --- | --- | --- | --- |
| ***NOTCH3*** | calcium ion binding, protein binding, enzyme binding | Endocrine resistance, Notch signaling pathway, Apelin signaling pathway, Th1 and Th2 cell differentiation, Thyroid hormone signaling pathway, Human papillomavirus infection, Pathways in cancer and Breast cancer | Cerebral Arteriopathy, Autosomal Dominant, With Subcortical Infarcts And Leukoencephalopathy, Type 1 and Myofibromatosis, Infantile, cancer |
| ***B4GALNT1*** | transferase activity and acetylgalactosaminyltransferase activity | Glycosphingolipid biosynthesis - ganglio series, Metabolic pathways | Spastic Paraplegia 26, Autosomal Recessive and Spastic Paraplegia 26 |
| ***GTF3C5*** | Involved in RNA polymerase III-mediated transcription. Integral, tightly associated component of the DNA-binding TFIIIC2 subcomplex that directly binds tRNA and virus-associated RNA promoters | RNA Polymerase III Transcription Initiation and Activated PKN1 stimulates transcription of AR (androgen receptor) regulated genes KLK2 and KLK3 | Endometrial cancer |
| ***TMEM106B*** | Lysosome localization, dendrite morphogenesis |  | Leukodystrophy, Hypo myelinating, 16 and Semantic Dementia |
| ***SMAP2*** | GTPase activator activity | Endocytosis | microsatellite instability oncogenesis |
| ***SNAPC2*** | DNA-binding transcription factor activity | Formation of HIV-1 elongation complex containing HIV-1 Tat and RNA Polymerase III Transcription Initiation | Glioblastoma |
| ***PTOV1*** | May activate transcription. Required for nuclear translocation of FLOT1. Promotes cell proliferation |  | prostate adenocarcinomas, tumorigenesis and progression of esophageal squamous cell carcinoma |
| ***MFAP2*** | fibronectin binding and fibrinogen binding | Canonical and Non-canonical Notch signaling and Notch signaling pathway | obesity, diabetes, osteopenia, head and neck squamous cell carcinoma, Costello Syndrome, gastric cancer |
| ***ZNF865*** | transcription coregulatory activity |  | Breast cancer |
| ***DDB2*** | ubiquitin-protein transferase activity and damaged DNA binding | Glioma and Metabolism of proteins | Xeroderma Pigmentosum, Complementation Group E and Xeroderma Pigmentosum Group E, Ovarian cancer, breast cancer, colon cancer |
